# Urothelial-lined perineal masses in hypospadias: clinical and pathological insights

**DOI:** 10.3389/fped.2026.1876663

**Published:** 2026-08-24

**Authors:** Enfu Huang, Chao Wang, Li Tao, Jie Zhu, Ting Zhang, Xiangming Yan, Xueming Zhu, Yun Zhou

**Affiliations:** 1 Department of Urology, Children's Hospital of Soochow University, Jiangsu, China; 2 Department of Surgery, Children's Hospital of Soochow University, Jiangsu, China; 3 Department of Pathology, Children's Hospital of Soochow University, Jiangsu, China

**Keywords:** anorectal malformations (ARMs), hypospadias, perineal mass, urothelium, von Brunn’s nests

## Abstract

**Introduction:**

To present a case series of the proximal hypospadias associated with perineal mass, which the perineal lesions were lined by urothelium containing Von Brunn's nests. A literature review was conducted to summarize the clinical and pathological features of similar cases.

**Methods:**

Two cases of proximal hypospadias with perineal mass treated at our institute were analyzed. Clinical presentation, diagnostic evaluation, surgical management, and histopathological findings are described. A literature review was performed in PubMed with the terms “(‘perineal mass’ OR ‘perineal anomaly’ OR ‘perineal’) AND (‘hypospadia’ OR ‘anorectal malformations’ OR ‘duplication’)”.

**Results:**

Both patients presented were detected by physical examination. One case had a normal anus, while the other had anorectal malformations (ARMs). Both cases showed normal Karyotype (46XY). Magnetic resonance imaging (MRI) demonstrated a soft tissue lesion that has not connection with or communication with adjacent organs. Both of then underwent mass excision and urethroplasty after cystoscopy, which revealed a normal bladder and posterior urethra. Pathological result of perineal mass suggested the histological structures of urothelium with von Brunn's Nests. There were totally 8 cases of hypospadia with perineal mass reported in the literature. Reported histological findings included 3 (37.5%) colonic structure, 2 (25%) lipoma, 2 (25%) accessory scrotum with hamartom and 1 (12.5%) sacrococcygeal teratomain.

**Discussion:**

Perineal mass with proximal hypospadias is an exceptionally rare anomaly. The histopathological founding of perineal mass lined by urothelium with von Brunn's nests has not been previously reported.

## Introduction

Congenital perineal masses are extremely rare and may occur either in isolation or in association with anorectal malformations (ARMs) and other urogenital anomalies (1). Although most reported cases are associated with ARMs, coexistence with proximal hypospadias is particularly uncommon. Reported perineal masses in this setting include intestinal duplication, lipoma, hamartoma, accessory labioscrotal fold, and other developmental lesions, reflecting considerable histopathological heterogeneity.

Here, we report two boys with proximal hypospadias, bifid scrotum, and a congenital perineal mass treated at a single institution. Histopathological examination demonstrated urothelial-lined lesions with prominent von Brunn's nests, a finding that, to our knowledge, has not been previously reported in association with proximal hypospadias. In addition to describing the clinical, surgical, and pathological characteristics of these patients, we performed a comprehensive review of the published literature and discuss the potential embryological mechanisms underlying this previously unrecognized entity.

## Materials and methods

This is an observational, noncomparative, retrospective study. We report two patients diagnosed as hypospadias with perineal mass, which confirmed the presence of von Brunn's nest hyperplasia, and managed in our institute. All patients in our series had clinical figures and pathological results. This study was approved by The Institutional Review Board.

### Case series

#### Case #1

**Figure 1:**
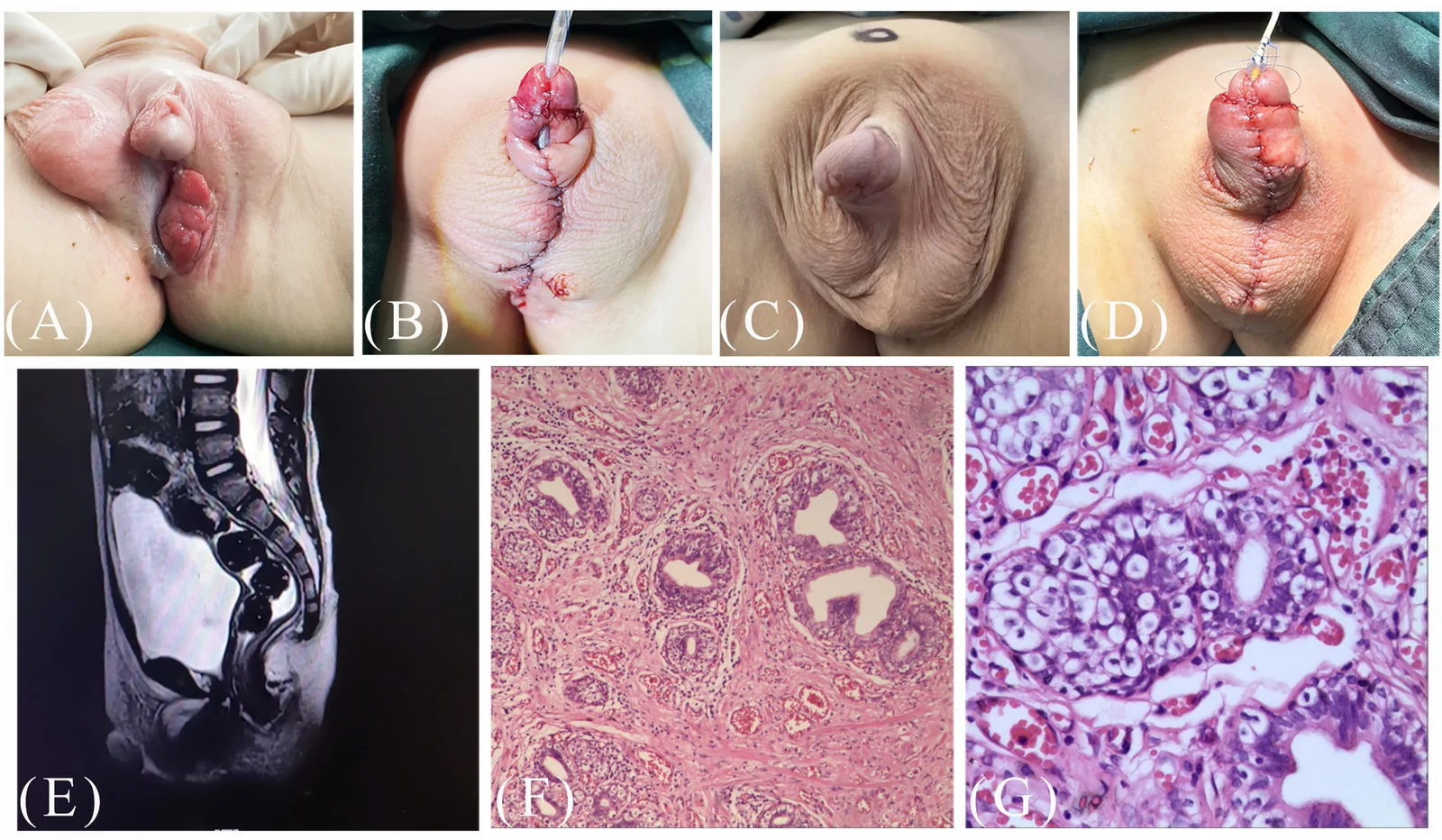
Preoperative and postoperative appearance, imaging examination, and HE staining of pathological sections in CASE 1. **(A)** Perineal mass, bifid scrotum, and hypospadias. **(B,D)** Postoperative appearance of first and second staged surgery. **(C)** Preoperative appearance of the second staged surgery. **(E)** Magnetic resonance imaging. **(F)** Urothelium with squamous metaplasia, von Brunn's nests extende into the superficial and deep lamina propria (100×). **(G)** von Brunn's nests (400×).

A 3-year-old boy was admitted to our department with an external perineal malformation. He was the second child of healthy, non-consanguineous parents, with no family history of congenital anomalies. The perineal lesion had been noted at birth. Physical examination revealed a 4 cm × 2 cm × 1 cm mucosal lesion located between the urethral meatus and the anus, associated with proximal hypospadias, bifid scrotum, and penoscrotal transposition (PST), while the anus was normally positioned (Figure 1A). Both testes were of normal size and had descended into the scrotum. Neonatal evaluation included hematologic testing, which showed a normal 46XY karyotype. Serum sex hormone levels and whole-exome sequencing (WES) were unremarkable. On admission, abdominal ultrasonography demonstrated normal kidneys and urinary tract. Pelvic magnetic resonance imaging (MRI) demonstrated the soft tissue protruding from the perineum without perineal fistula and ruled out urethral duplication (Figure 1D). Cystoscopy revealed a normal bladder, no posterior urethral pathology, including valves or prostatic utricle, was detected. The lesion was recognized in the neonatal period. As it did not interfere with micturition or defecation, surgical excision combined with hypospadias repair between 6 and 12 months of age was recommended. Nevertheless, the parents chose to postpone surgery for their own concern. The mass remained stable during an otherwise uneventful observation period. The child underwent the first stage of repair at age three, consisting of perineal mass excision, penile straightening by transecting the urethral plate, and partial distal urethral reconstruction using a modified Ulaanbaatar technique (Figures 1B,C) (2). Although the parents elected to postpone the second stage, the reconstructed urethral plate remained in good condition during the prolonged interval. The second-stage urethroplasty was completed at 6 years of age, together with correction of PST (Figure 1D).

#### Case #2

**Figure 2:**
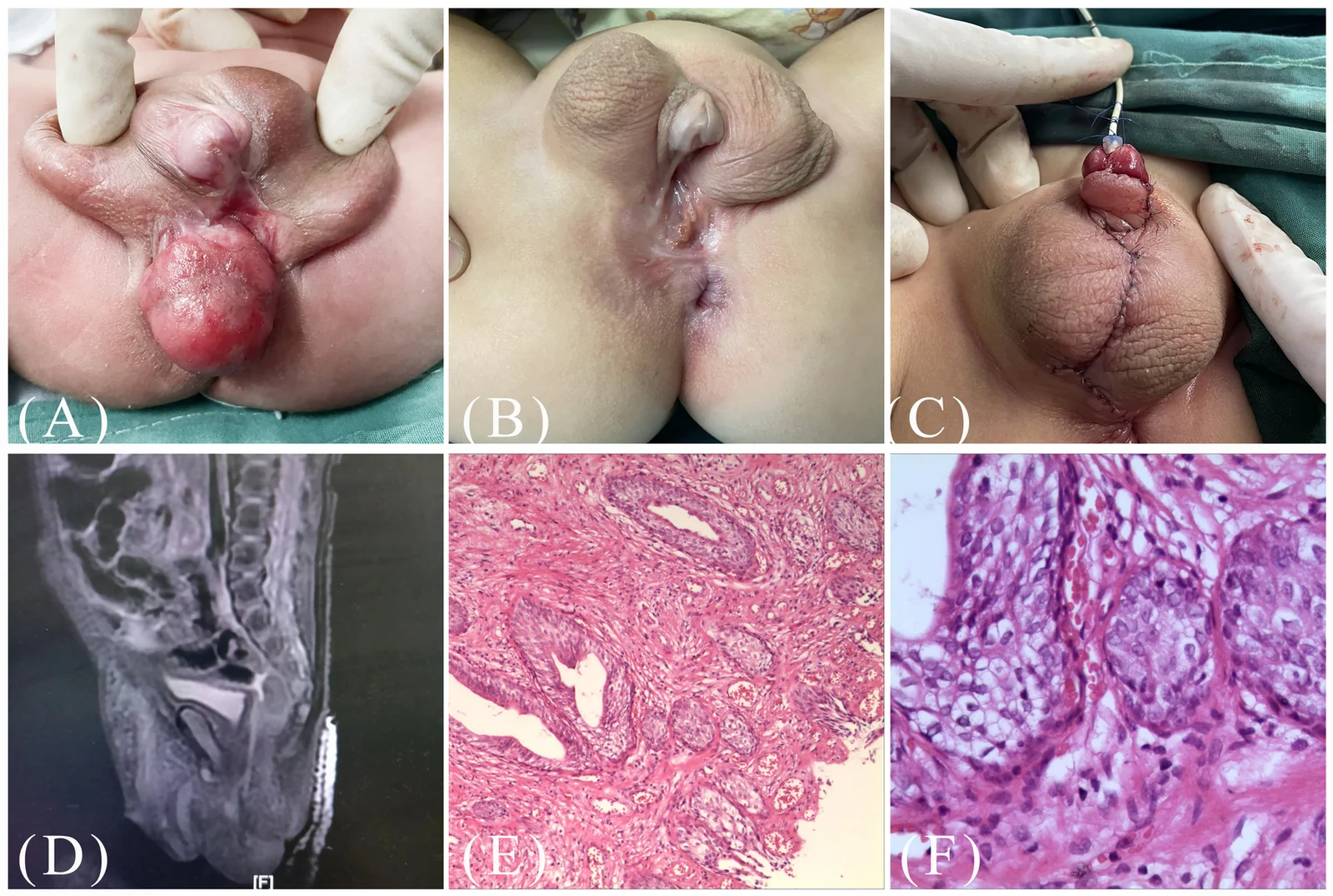
Preoperative and postoperative appearance, imaging examination, and HE staining of pathological sections in CASE 2. **(A)** Perineal mass, bifid scrotum, and hypospadias. **(B)** 1-year-old's appearance, hypospadias and ectopic anus. **(C)** Postoperative appearance. **(D)** Magnetic resonance imaging. **(E)** Urothelium with squamous metaplasia, von Brunn's nests extended into the superficial and deep lamina propria (100×). **(F)** von Brunn's nests (400×).

A male neonate born at 36 weeks of gestation with a birth weight of 2,920 g presented with a smooth, soft, spherical perineal mass, hypospadias and an anteriorly placed anus with stenosis was referred to our center. Physical examination revealed a 4 cm × 3 cm perineal mass arising from the caudal aspect of the right scrotum, also with bifid scrotum, and PST. A stenotic anal opening was identified at the inferior aspect of the mass (Figure 2A). Karyotype was 46XY, and hormonal, genetic, and endocrine evaluations were normal. Abdominal ultrasonography showed no urogenital abnormality except the proximal hypospadias with bifid scrotum, penoscrotal transposition. Preoperative MRI confirmed a low ARM and excluded the presence of any urethrorectal fistula (Figure 2D). At 16 days of age, he underwent excision of the perineal mass and cutback anoplasty, which relieved the stenosis (Figure 2B). At 1 year of age, the anorectum was mobilized and repositioned into the center of the sphincter complex under muscle stimulation guidance, and his hypospadias was repaired in a single stage using a modified Koyanagi technique (Figure 2C) (3). Cystoscopy was performed before the second surgery, and no posterior urethral pathology was detected, including valves or prostatic utricle.

### Literature review

The PubMed database was queried using the key terms “(‘perineal mass’ OR ‘perineal anomaly’ OR ‘perineal’) AND (‘hypospadia’ OR ‘anorectal malformations’ OR ‘duplication’)” to identify published cases of congenital perineal mass with hypospadia or other complex perineal malformations. Six reports on perineal mass, which were similar in appearance to our case, associated with hypospadias were identified and reported (Table 1) (4–10).

**Table 1:** Summary of all 10 cases of perineal masses with hypospadias in our institute and literature series.

Author (year)	Age	Sex	Urethra abnormalties	Other abnormalities	Surgical treatment (time)	Histology
Current study	3 Years	M	Hypospadias	Bifid scrotum PST	Excision; Urethroplasty (3 years)	Urothelium
Current study	Neonate	M	Hypospadias	Bifid scrotum PST Imperforate anus	Excision; PSARP (16 days); Urethroplasty; Penile & scrotal anaplasty (1 year)	Urothelium
Thomas et al. (2002) (4)	1 Month	M	Hypospadias	Hemiperineal defect Exposed mucosa Bifid scrotum PST	Excision; Diverting colostomy; Urethroplasty; Penile & scrotal anaplasty (UK)	Sacrococcygeal teratoma rupture
Ramzisham et al. (2005) (5)	Neonate	M	Hypospadia	Bifid scrotum PST Imperforate anus Rectoperineal fistula	Excision; PSARP; (5 months) Urethroplasty; Penile & scrotal anaplasty (UK)	AS; Hamartoma
Wester et al. (2006) (6)	3 Years	M	Hypospadias	Rectourethral fistula	Excision; Colostomy; PSARP; (20 months) Urethroplasty (3 years)	Lipoma
Wester et al. (2006) (6)	Neonate	M	Hypospadias	A high anorectal malformation PST	Excision; PSARP; (4 months) Urethroplasty; (UK) Umbilical ACE-stoma (11 years)	Lipoma
Sun et al. (2011) (7)	Neonate	M	Hypospadias	Bifid scrotum PST	Excision; Urethroplasty; Penile & scrotal anaplasty (1 months)	Rectal duplication
Danish et al. (2020) (8)	Neonate	M	Hypospadias	Bifid scrotum PST	Excision. (18 months)	Colorectal Hamartoma
Wolffenbuttel and Sloots (2020) (9)	7 Years	M	Hypospadias Urethral duplication	Bifid scrotum PST	Excision; Urethroplasty; Penile & scrotal anaplasty (7 years)	Colonic
Wang et al. (2021) (10)	UK	M	Hypospadia	Rectoperineal fistula Incomplete caudal duplication syndrome	Excision; PSARP; Urethroplasty (UK)	AS; Mesenchymal hamartoma

PST, penoscrotal transposition; PSARP, posterior sagittal anorectoplasty; AS, accessory scrotum; ACE, antegrade colonic enema; UK, unknow.

## Results

### Surgical outcome

Both cases showed favorable postoperative recovery, maintaining good cosmetic appearance, stable urinary function, and no major complications noted during follow-up.

#### Case #1

At 1 year of follow-up, the incision healed uneventfully with no evidence of wound dehiscence or urethrocutaneous fistula. The patient voided in a standing position, and uroflowmetry demonstrated a Qmax = 9.43 mL/s (Vvol = 55.18 mL), and he remains under regular follow-up.

#### Case #2

At 3 years of follow-up, the penile appearance was satisfactory with no stenosis or meatal retraction. Post-anoplasty assessments at 1, 3, and 6 months were normal, and routine anal dilatation was not required. Uroflowmetry demonstrated a Qmax = 7 mL/s (Vvol = 47 mL), indicating a favorable urinary stream. Annual follow-up is ongoing.

### Pathological findings

Histologically, both lesions shared a similar feature: lining by a single or stratified urothelium with extensive squamous metaplasia (Figures 1E, 2F), filled with foci of acute inflammation, surface mucosal denudation, and granulation tissue formation, resembling von Brunn's nest hyperplasia or cystitis cystica glandularis. These epithelial nests were characteristically surrounded by concentric fibrosis; scattered smooth muscle bundles were also noted. The surrounding stroma was richly vascular accompanied by variable edema and chronic inflammation, without nuclear atypia (Figures 1F, 2G).

### Literature review

We performed a literature search for cases of hypospadia with perineal mass. A total of 8 cases were identified in the published literature (Table 1). Among them, 5 patients (62.5%) presented with PST and bifid scrotum, 4 (50%) had associated ARMs, and 1 (12.5%) had urethral duplication. Excluding two reports that did not specify the timing of surgery, all patients with ARMs underwent surgical treatment before the age of 2-year-old. In these cases, excision of the perineal mass was performed concurrently with anorectal reconstruction, while further surgery for hypospadias correction was postponed to a later stage.

Pathological results showed the colonic structure in 3 cases (37.5%), lipoma in 2 cases (25%), accessory scrotum with hamartoma in 2 cases (25%) and sacrococcygeal teratomain in 1 case (12.5%).

## Discussion

Hypospadias associated with a perineal mass represents an exceptionally uncommon and complex defect of perineal development and may coexist with ARMs or other urogenital anomalies. Because of the rarity and heterogeneity of the reported lesions, both diagnosis and treatment planning require particular caution and individualized decision-making. For patients without obstruction, emergency surgery is generally unwarranted. Instead, comprehensive preoperative evaluation is essential, including ultrasonography and MRI, to define the extent of the lesion, its relationship with adjacent structures (urethra, bladder, genitalia, and rectum), and to exclude sinus tracts or fistulas (1, 11). In the setting of ARMs, characterization of the specific subtype and assessment for obstruction remain essential determinants of surgical strategy, even when urgent decompression is required.

The decision to excise a perineal mass during ARM repair depends on multiple factors, including its size, depth, relationship with the sphincter complex, and potential interference with the intended neoanal site (12). In our Case 2, the patient presented in the neonatal period with an anteriorly placed anus and anal stenosis, prompting early surgical intervention. Because the patient was a newborn and prolonged anesthesia in early infancy has been associated with potential neurodevelopmental risks, a limited approach was selected. Preoperative imaging confirmed that the perineal mass had no communication with adjacent structures, allowing cutback anoplasty with mass excision —completed in 60 min—to provide effective relief while minimizing anesthetic exposure. This staged approach prioritized safety and preserved tissues for later reconstruction.

Most published cases similarly report mass excision in conjunction with ARM repair once anatomy is clarified. In contrast, the timing and modality of hypospadias reconstruction remain highly individualized. The absence of a standardized operative algorithm reflects the need to account for multiple anatomical and patient-specific variables, including glans size, deficiency of the urethra, degree of curvature, quality of urethral plate, available reconstructive tissue and surgeon expertise.

In our series, anatomical differences between patients led to divergent surgical choices. In Case 1, several anatomical factors supported a staged repair. Although the patient had favorable penile and glans development (glans width 15 mm), the required length of urethral reconstruction was substantial at 62 mm—a magnitude that significantly increases the risk of urethral stricture, if attempted in a single stage. This strategy proved advantageous, as the second procedure ultimately required reconstruction of only a 40 mm segment. In contrast, Case 2 presented with a markedly smaller glans (10 mm) but a shorter urethral defect (42 mm). The unfavorable glans anatomy increased the risk of dehiscence in a staged approach, whereas the relatively shorter urethral deficiency supported the feasibility of a single-stage reconstruction. Accordingly, a modified Koyanagi repair was selected.

Therefore, these cases highlight a central principle: in the rare triad of proximal hypospadias, perineal mass, and ARMs, optimal management depends on precise delineation of anatomical relationships and tailored operative planning. Rigorous preoperative evaluation enables a coherent, function-preserving strategy that avoids compromising either urinary or anorectal reconstruction.

The histological diagnoses of perineal masses described previously include vascular malformations, lipomas, teratomas, rectal duplications, and accessory labioscrotal folds (ALF) in females or accessory scrotum (AS) in males. Clinical manifestations are often nonspecific because of the intimate anatomical relationships within the perineum. Although many lesions exhibit normal-appearing skin, histopathology may reveal a wide variety of tissues, including bone, muscle, glandular epithelium, dermal structures, or respiratory epithelium (11, 13). Soft, reddish perineal masses reported in the literature have often been diagnosed as rectal duplication, hemangioma, hamartoma, or accessory labioscrotal/scrotal tissue, with corresponding histological diversity.

The pathogenesis of perineal masses is not fully understood. Embryologically, the perineum arises from the urorectal septum, which divides the cloaca into the ventral urogenital sinus and dorsal anorectal canal (14). The cloacal and urethral plates are derived from endoderm. The urethral plate undergoes endodermal tubularization and subsequently fuses with ectoderm at the glans to form the external urethral meatus (15). This shared developmental origin explains the frequent coexistence of urogenital and anorectal anomalies in patients with perineal masses. Accordingly, abnormal proliferation or partitioning of the urorectal septum may result in the misplacement of endoderm-derived tissue—gastrointestinal, respiratory, or urinary—within the perineum.

Our literature review confirmed that perineal masses arising from urorectal septal maldevelopment display diverse clinical and histological features. Most reported lesions consist of intestinal or other gastrointestinal-type tissues, independent of associated ARMs. In contrast, although the lesions in our two patients were initially suspected to represent rectal duplication or vascular tumors, histopathology demonstrated exclusively urothelial components, including von Brunn's nests, cystitis cystica, and cystitis glandularis. To our knowledge, purely urothelial-derived perineal lesions have not been previously described.

Von Brunn's nests are considered as normal variants of urothelium. They represent benign, reactive proliferations of the urothelium characterized by invaginations of the surface epithelium into the lamina propria, forming well-circumscribed nests of urothelial cells. They are considered a normal histological variant and are present in up to 89%–95% of normal bladder mucosa in autopsy series. These nests are commonly associated with chronic irritation, inflammation, infection, or mechanical stimuli, such as urinary tract infections, indwelling catheters, or bladder outlet obstruction (16). They have also been described in patients with bladder exstrophy–epispadias, further supporting the concept that these lesions represent a reactive rather than neoplastic process (17).

As described in the literature, von Brunn's nests lack nuclear atypia, mitotic activity, stromal desmoplasia, and architectural disorder—features distinguishing them from malignant entities such as the nested variant of urothelial carcinoma. Although florid proliferations may mimic inverted or nested urothelial carcinoma, the preservation of polarity, uniform growth depth, and absence of invasion confirm a benign process.

In our cases, histologic examination demonstrated classic benign features without evidence of neoplastic transformation. The combination of urothelial lining, von Brunn's nests, and cystitis cystica/glandularis strongly indicates that the lesions were urothelial in origin, rather than derived from colorectal or mesenchymal elements, which are more frequently described in perineal lesions.

The histopathological findings in both of our patients closely resemble those reported in bladder exstrophy polyps, including urothelial lining with extensive squamous metaplasia, von Brunn's nests, cystitis cystica or glandularis-like changes and concentric stromal fibrosis (18). Such lesions are generally considered a reactive proliferative process induced by chronic exposure and persistent mucosal irritation rather than a true neoplasm. The remarkable histological similarity raises the possibility that the perineal lesions in our patients may share a common pathogenic mechanism despite their different anatomical locations.

During normal embryogenesis, the urethral plate and bladder mucosa share a common endodermal origin (19). Between approximately 8 and 19 weeks of gestation, the urethral groove normally extends from the pelvic urethra into the future glans penis, terminating just proximal to the glanular tip. During this period, progressive urethral tubularization and fusion of the urethral folds result in complete closure of the urethral groove, while the prepuce subsequently develops to fully cover the newly formed urethra (20). In severe proximal hypospadias, failure of urethral tubularization leaves the urethral plate persistently exposed beyond this normal developmental window. This primitive endoderm-derived urothelium may remain chronically exposed from late fetal life and throughout postnatal life to urine, environmental irritation, and recurrent inflammation.

The histopathological resemblance between our cases and bladder exstrophy polyps provides a plausible framework for understanding the origin of these lesions. Similar to the chronically exposed bladder mucosa in bladder exstrophy, persistent exposure of the urethral plate may trigger reactive urothelial proliferation characterized by von Brunn's nests, cystitis cystica, and squamous metaplasia, ultimately resulting in a polypoid perineal lesion. Although this hypothesis cannot be confirmed by the present cases alone, the consistent pathological findings in both patients support chronic inflammatory proliferation of exposed endoderm-derived urothelial tissue as a plausible pathogenic mechanism.

Alternatively, the lesion may represent heterotopic endoderm-derived urothelial tissue misplaced during cloacal partitioning or urethral plate development. The lower genitourinary tract and anorectum develop between the 15th and 18th gestational weeks, while labioscrotal swellings undergo migration and fusion by approximately 12 weeks of gestation (21). During this critical developmental period, coordinated endodermal–mesodermal interactions are required for normal formation of the urethra, perineum, and external genitalia. Aberrant displacement of endoderm-derived urothelial progenitor cells, together with abnormal mesenchymal patterning, could result in ectopic urothelial tissue within the perineum. Similar embryological misallocation has been proposed for several congenital perineal lesions associated with bifid scrotum, hypospadias, and anorectal malformations, supporting this developmental hypothesis (22).

A third possibility is that the lesion represents a duplicated remnant of the urethral plate rather than simple heterotopia. Intraoperatively, a distinct tissue plane was consistently identified between the mass and the native urethral plate, arguing against direct inflammatory expansion of the urethral plate itself. An accessory urethral plate that failed to canalize or establish communication with the urinary tract could theoretically persist as an isolated endoderm-derived urothelial remnant. Prolonged exposure of this duplicated urothelial tissue from late gestation onward may lead to reactive urothelial proliferation.

At present, none of these hypotheses can be definitively confirmed. A major limitation is the lack of comparative histological evidence from the adjacent urethral plate or corpus spongiosum, as biopsy of these normal structures would have been neither ethically justifiable nor clinically indicated. Therefore, the proposed developmental relationship between the perineal mass and the native urethral plate remains inferential rather than directly demonstrated. In addition, no immunohistochemical or molecular lineage analyses were performed to further investigate the developmental origin of the lesions. The benign urothelial architecture, absence of cytological atypia, and striking histopathological resemblance to reactive lesions described in bladder exstrophy all point to chronic inflammatory proliferation of endoderm-derived urothelium as the most likely explanation. Nevertheless, the complexity of perineal embryogenesis and the intraoperative identification of a discrete tissue plane leave open the possibility of heterotopic urothelial tissue or a duplicated urethral plate remnant. The consistency of pathological findings across the two independent patients suggests that urothelial perineal masses may represent a distinct, previously undescribed entity associated with severe proximal hypospadias. Further studies—incorporating comparative histology, embryological investigation, immunohistochemistry, and molecular characterization—would be necessary to clarify their developmental origin.

Given the urothelial origin of these lesions, long-term clinical surveillance appears prudent. Although complete excision was achieved in both patients and the histological findings were most consistent with reactive inflammatory proliferation, the risks of recurrence and long-term biological behavior remain unknown because no comparable cases have been reported. Chronically exposed urothelium, as exemplified by bladder exstrophy, is associated with reactive proliferative changes and a markedly increased risk of subsequent malignancy, suggesting that prolonged urothelial irritation may have important biological consequences (23, 24). While such risks cannot be directly extrapolated to the present lesions, they underscore the importance of continued clinical follow-up. Periodic clinical examination, together with urinary tract assessment when clinically indicated, may facilitate early detection of recurrence or obstructive complications. As additional cases accumulate, the optimal surveillance strategy can be better defined.

## Limitation

Several limitations of this study deserve mention. The retrospective design and the very small sample size reflect the extreme rarity of perineal masses associated with proximal hypospadias; inevitably, the limited number of cases restricts the generalizability of our findings and precludes meaningful statistical analysis. Follow-up was confined to early childhood, which prevents a comprehensive view of the natural history of these urothelial-derived lesions and their possible long-term functional consequences. Larger, multicenter studies with extended follow-up are therefore needed to validate our observations and to clarify the pathogenesis of this previously undescribed entity.

Although both patients achieved satisfactory early cosmetic and urinary outcomes, prolonged follow-up through adolescence and adulthood remains essential, because several key functional parameters cannot be adequately assessed during childhood. Pubertal evaluation should specifically address erectile function, ejaculatory function, and patient-reported sexual outcomes. Recent work has emphasized the role of bulbospongiosus muscle reconstruction in restoring penile support, improving post-micturition emptying, and facilitating normal ejaculatory function in patients with proximal hypospadias (25, 26), suggesting that the anatomical repair performed in childhood may carry important implications for adult sexual function.

Beyond uroflowmetry, long-term assessment of lower urinary tract function should encompass a more comprehensive panel: urinary continence, bladder neck competence, post-void residual urine, and, when clinically indicated, urodynamic studies. Such evaluation could provide valuable insight into the functional maturation of the reconstructed lower tract. Finally, it remains unknown whether the extensive developmental abnormalities involving the proximal urethra and perineum predispose these patients to subtle bladder neck dysfunction later in life—a question that clearly warrants further investigation.

## Conclusion

Perineal masses associated with hypospadias are exceedingly rare, and their embryogenesis remains complex and poorly understood. Our two cases broaden the pathological spectrum of such lesions by demonstrating that urothelial-derived reactive proliferations can occur ectopically in the perineum. Recognizing this entity is clinically important, as it may mimic rectal duplication or mesenchymal tumors but differs markedly in origin and management. These cases further emphasize the need for precise anatomical assessment and individualized surgical planning—particularly regarding the timing and technique of urethral reconstruction—to achieve optimal outcomes. Lifelong surveillance remains essential given the uncertain long-term behavior of reactive urothelial proliferations.

## Data Availability

The original contributions presented in the study are included in the article/Supplementary Material, further inquiries can be directed to the corresponding author.
